# High prevalence of potential biases threatens the interpretation of trials in patients with chronic disease

**DOI:** 10.1186/1741-7015-9-73

**Published:** 2011-06-13

**Authors:** Daniela Vollenweider, Cynthia M Boyd, Milo A Puhan

**Affiliations:** 1 Division of General Internal Medicine, Department of Medicine, Johns Hopkins University, Baltimore, MD, USA; 2 Division of Geriatric Medicine and Gerontology, Department of Medicine, Department of Medicine, Johns Hopkins University, Baltimore, MD, USA; 3 Horten Centre for patient-oriented research, University of Zurich, Zurich, Switzerland; 4 Department of Epidemiology, Johns Hopkins Bloomberg School of Public Health, Baltimore, MD, USA

## Abstract

**Background:**

The complexity of chronic diseases is a challenge for investigators conducting randomized trials. The causes for this include the often difficult control for confounding, the selection of outcomes from many potentially important outcomes, the risk of missing data with long follow-up and the detection of heterogeneity of treatment effects. Our aim was to assess such aspects of trial design and analysis for four prevalent chronic diseases.

**Methods:**

We included 161 randomized trials on drug and non-drug treatments for chronic obstructive pulmonary disease, type 2 diabetes mellitus, stroke and heart failure, which were included in current Cochrane reviews. We assessed whether these trials defined a single outcome or several primary outcomes, statistically compared baseline characteristics to assess comparability of treatment groups, reported on between-group comparisons, and we also assessed how they handled missing data and whether appropriate methods for subgroups effects were used.

**Results:**

We found that only 21% of all chronic disease trials had a single primary outcome, whereas 33% reported one or more primary outcomes. Two of the fifty-one trials that tested for statistical significance of baseline characteristics adjusted the comparison for a characteristic that was significantly different. Of the 161 trials, 10% reported a within-group comparison only; 17% (n = 28) of trials reported how missing data were handled (50% (n = 14) carried forward last values, 27% (n = 8) performed a complete case analysis, 13% (n = 4) used a fixed value imputation and 10% (n = 3) used more advanced methods); and 27% of trials performed a subgroup analysis but only 23% of them (n = 10) reported an interaction test. Drug trials, trials published after wide adoption of the CONSORT (CONsolidated Standards of Reporting Trials) statement (2001 or later) and trials in journals with higher impact factors were more likely to report on some of these aspects of trial design and analysis.

**Conclusion:**

Our survey showed that an alarmingly large proportion of chronic disease trials do not define a primary outcome, do not use appropriate methods for subgroup analyses, or use naïve methods to handle missing data, if at all. As a consequence, biases are likely to be introduced in many trials on widely prescribed treatments for patients with chronic disease.

## Background

Previous studies have shown that the quality of reporting of important aspects of randomized clinical trials (RCTs) is often poor [1-6]. These studies have indicated that a substantial proportion of RCTs might be at high risk for confounding, selection and information biases because they were not designed optimally to minimize threats to internal validity. For example, the method of randomization was often not reported, so that it was not clear if the RCTs actually controlled for confounding by correct randomization, or concealment of random allocation was not described, which may have put the studies at risk for selection bias. Many trial reports also lacked details about the method of masking of patients, treatment providers or those ascertaining the outcomes. The introduction of CONSORT (CONsolidated Standards of Reporting Trials) improved the quality of reporting of clinical trials, but there is still a large proportion of RCTs that are of uncertain internal validity and value for informing clinical practice [7].

The complexity of chronic diseases represents additional challenges to investigators conducting randomized trials. Even if a trial scores high on the standard criteria for assessing the risk of bias (method of randomization, concealment and masking), it may still not provide high-quality data to answer a research question. For example, patients with chronic diseases form heterogeneous study populations, so that it may be more difficult to control for confounding. In addition, chronic disease trials often have longer follow-up durations so that the risk for low treatment adherence and missing data increases, which may lead to selection bias [8]. Finally, there is often a great variety of potential outcomes available including mortality, various clinical events, patient-reported outcomes, and surrogate outcomes. As a consequence, some RCTs may not explicitly define a primary outcome, even though this is important to calculate the required sample size and to avoid selective outcome reporting [9]. In addition, although not choosing a primary outcome does not directly lead to biased results, it may lead to study designs that do not optimally control for confounding for some outcomes. For example, if 5-year mortality is the primary outcome in a trial, the confounders that should be taken into consideration for the study design are likely to be different from a trial where quality of life after 1 year is the primary outcome [9,10]. Restriction of the study population and pre-stratification, two powerful tools to control for confounding, would be designed differently depending on whether the primary outcome is 5-year mortality or 1-year quality of life. Finally, treatment effects may vary across heterogeneous study populations, and subgroup analyses should be appropriately designed, conducted and reported to avoid spurious findings [11,12].

We were interested in the proportion of trials that are at risk of biases caused by these aspects of trial design and analysis. To evaluate such sources of bias, which t have received less attention in studies on prevalent and high-burden chronic diseases, our aim was to assess these aspects in trials that investigated the effects of widely prescribed drug and non-drug treatments.

## Methods

### Study design

We conducted a survey of trial reports on widely prescribed drug and non-drug trials in patients with four common chronic diseases: chronic obstructive pulmonary disease (COPD), heart failure, stroke and type 2 diabetes mellitus (T2DM).

### Study selection

We decided to focus on diseases associated with high morbidity and mortality for patients and with significant healthcare expenditure [13-15]. We hypothesized that methodological deficiencies of trials that provided evidence on treatments for such diseases could bias effect estimates and, therefore, clinical practice. We focused on widely prescribed drug and non-drug therapies for which we wanted to have comprehensive sets of trials as identified by systematic reviews, each addressing a specific research question. Therefore, we based the selection of RCTs on 11 Cochrane Reviews that systematically identified and summarized RCTs on the effectiveness of diuretics, metformin, anticoagulants, long-acting β agonists alone or in combination with inhaled corticosteroids, lipid-lowering agents and the non-drug interventions of exercise and diet for each of the four diseases [16-26]. We included all available reviews in these fields except for the stroke literature, where several other reviews about other drug and non-drug treatments exist. The search strategy and eligibility criteria have been described in these Cochrane reviews. We retrieved the main reports of included RCTs, and also retrieved additional papers that described the methods of these trials. We assessed only the reporting of trials and not their conduct because the protocols or internal reports were not available to us. We did not consider abstracts and unpublished data used in the Cochrane reviews because they could not provide the level of detail that we needed, and we excluded 22 trials (out of 183) for that reason. The bibliography of excluded trials is available on request.

### Data extraction

Before systematically extracting data from each trial, we developed a codebook that provided a detailed description of the information to be extracted and how to score it. We pilot tested the data-extraction forms and the codebook on a random sample of 10 articles. All data were then extracted by one reviewer into an online database, and checked by at least one other reviewer. Disagreements were discussed and resolved.

#### Definition of primary outcome

We recorded whether there were one or more clearly defined primary outcomes. Beside guiding interpretation [27] and sample size calculations, defining a primary outcome is important for designing a trial that minimizes confounding indirectly by the use of restriction (exclusion criteria that eliminate some levels of characteristics that may act as confounders), pre-stratification for prognostically important variables, and the collection of data for potential statistical adjustment. Confounders can be profoundly different depending on the primary outcome (for example, mortality versus quality of life) [9]. Possible answer keys for data extraction included 'Yes, clearly defined' if the authors made an explicit distinction between the primary and secondary outcomes, and 'No' if they did not make distinction between primary and secondary outcomes. For those papers that did describe a primary outcome, we also recorded whether the primary outcome was only one measure (for example, percentage of forced expiratory volume in one second predicted at 1 year of follow-up) or whether multiple measures were considered as primary outcome (for example, different individual measures, or one measure at different time points at which treatment effect may differ).

#### Between- and within-group comparisons

We recorded whether or not there were only within-group comparisons reported and no reporting of between-group comparisons ('Yes', 'No' or 'Unclear'). Within-group comparisons are a comparison of baseline and follow-up measurements within each treatment group, rather than an effect estimate for a randomized comparison between groups. Based on a within-group comparison, it is not possible to tell whether a change was caused by the intervention or by some other factor.

#### Comparison of baseline characteristics and decisions to adjust for baseline imbalances

We recorded whether a between-group comparison of baseline characteristics was made to assess significant differences (for example, *P *values in Table 1). This is not a particularly useful comparison, because it tests the null hypothesis that treatment groups are not different, even though we know that through randomization the null hypothesis is true [28-30]. Even more importantly, such testing may misguide the statistical analysis as investigators may inappropriately use these differences to over- or under-adjust for potential confounders, even though significant differences at baseline occur by chance in 5% of the variables tested. Indeed, confounding of observed treatment effects may result if certain characteristics are not well balanced and are thus associated with treatment exposure, or if they influence the outcome but are not a result of treatment exposure (intermediates). However, the decision for or against considering a variable to be a confounder should not be made based on testing for statistical significance but rather on prior evidence and/or biological rationale. Therefore, we distinguished between 'Yes, reporting of *P *values and/or the term statistical significance', 'Yes, significant' (which may refer to a statistical analysis but also to a clinically relevant difference), 'No' and 'Does not apply' in the case of crossover studies. For those papers in which the investigators tested for baseline differences, we assessed what actions were taken as a consequence of testing. We recorded if the authors adjusted for a significant difference in a baseline characteristic or mentioned it in the Discussion section. If they did not find any significant differences, we assessed if they adjusted for large-magnitude differences in baseline characteristics irrespective of significant tests, or adjusted for a potential confounder unrelated to baseline characteristics.

#### Missing data

Missing data occur in almost any study for some outcomes or covariates [10]. We were interested in how chronic disease trials handled missing data because of the potential bias on treatment effects. If data are missing at random and non-differentially in different treatment groups, effect estimates are, in the best case, still valid although less precise. However, if patients drop out of a trial (for example, because of adverse effects) and do not provide outcome data, a selection bias may occur if these patients are dropped from the analyses or censored [8]. In addition, if data for confounders are missing, the statistical adjustment for potential confounding is compromised. We assessed whether or not the approach to the handling of missing data was reported ('Yes', 'No' and 'Unclear') and recorded the corresponding methods (for example, last value carried forward, imputation of fixed values such as mean or multiple imputation). If the authors reported the missing data, but dropped those participants from analysis, we recorded it as a complete case analysis.

#### Intention-to-treat analysis

We also assessed whether or not an intention-to-treat (ITT) analysis was reported. An ITT analysis is an analysis based on the initial treatment intent, not on the treatment eventually administered. Changing a patient from their assigned treatment arm to another arm during the trial and/or dropping some patients from the analysis also leads to selection bias. We considered an analysis to be ITT if the authors explicitly described the analysis as such, or if the numbers of patients included in the analysis corresponded exactly to those randomized to the respective treatment groups [31].

#### Reporting of point estimates and measures of precision

To interpret treatment effects, trial reports should include point estimates, confidence intervals (CIs) and *P *values. *P *values alone do not suffice to interpret the results of trials because they are influenced by both sample size and effect size. We registered whether trial reports included *P *value only, 95% CI only, both, or neither.

#### Subgroup analyses to investigate heterogeneity of treatment effects

Finally, we assessed how often and in what way subgroup analyses were performed, using recently described criteria [11,12]. We defined a subgroup analysis as the analysis of an effect that varied (or not) depending on different levels of a variable measured before randomization. We assessed whether this occurred by using 'Yes' and 'No'. For those trials that did report one or more subgroup effects, we recorded whether just one, a small number (two to five) of or a large number (more than five) of subgroup effects were reported. We recorded the proportion of trials using an interaction term to compare treatment effects across various other factors and whether the interaction terms were statistically independent of other subgroup effects. In addition, we assessed whether or not subgroup effects were consistent across closely related outcomes. We also assessed if the authors had specified a hypothesis for why a subgroup effect could be present, based on evidence from the literature or biological plausibility, and whether they pre-specified the direction of the subgroup effect.

### Statistical analysis

We used descriptive statistics to summarize our findings across the entire set of trials, and stratified by disease, type of treatment (drug versus non-drug), time of publication (before and after 2001, when many journals adopted the CONSORT statement) and the impact factor of the journal in which the reports were published (per unit increase of the 2009 impact factor), which we used as an indirect measure for the overall quality of a trial. We used simple and multiple logistic regression analysis to detect the association of the sources of bias we assessed with type of treatment, time of publication and the impact factor of the journal. For analyses with time of publication as a covariate, we restricted the analysis to trials published in or after 1990, because initiatives to improve trial reporting, such as CONSORT, did not start before 1990. All analyses were conducted with Stata for Windows (version 10.1; Stata Corp., College Station, TX, USA).

## Results

Based on the 183 studies included in 11 Cochrane reviews, we were able to include 161 RCTs published between 1966 and 2009. The distribution of the type of diseases evaluated in the reports was as follows: COPD (n = 49), heart failure (n = 31), T2DM (n = 48), stroke (n = 33). Nearly half (43%; n = 70) were drug trials, which assessed long-acting β agonists (n = 21), lipid-lowering agents (n = 7), metformin (n = 26), diuretics (n = 12) and oral anticoagulants (n = 4), while the remainder (57%; n = 91) assessed were non-drug interventions such as rehabilitation (n = 69) and exercise and/or diet (n = 22).

### Sources of bias, overall and stratified for type of disease and type of intervention

Testing for statistical significance of baseline characteristics was not reported by 68% (n = 110) of all trials (Table 1). Of the 51 papers that did reported testing for statistical significance of baseline characteristics, 38% (n = 20) found at least one characteristic with a significant difference. Of these twenty papers, two (10%) had results adjusted for the characteristic; four (20%) had the difference in baseline characteristics discussed in the Discussion section, and one (5%) (in which no significant difference in baseline characteristics was found) had the results adjusted for a characteristic measured at baseline because the difference between groups was considered to be large. A between-group comparison was reported in 90% of the 161 trials (n = 145), while 10% reported only a within-group analysis. An ITT analysis was reported in 42% (67 trials).

**Table 1:** Reporting of aspects of trial design, conduct and analysis: Stratified by diseases and types of Intervention

Interventions	All	Drug		COPD		Heart failure		Diabetes		Stroke	
			**Non-drug**	**Drug**	**Non-drug**	**Drug**	**Non-drug**	**Drug**	**Non-drug**	**Drug**	**Non-drug**

**Number of trials**	161	70	91	20	29	12	19	26	22	12	21
**Reporting of between- group comparisons, % (n)**	90% (145)	90% (63)	89% (81)	100% (20)	93% (27)	67% (8)	95% (18)	92% (24)	82% (18)	92% (11)	86% (18)
**Primary outcome, % (n)**	33% (53)	50% (35)	20% (18)	70% (14)	7% (2)	17% (2)	21% (4)	42% (11)	36% (8)	67% (8)	19% (4)
**Primary outcome is only one measure, % (n)**	21% (34)	31% (22)	13% (12)	35% (7)	0% (0)	8% (1)	16% (3)	27% (7)	32% (7)	58% (7)	10% (2)
**No statistical comparison of baseline characteristics, % (n)**	68% (110)	79% (55)	60% (55)	85% (17)	59% (17)	83% (10)	68% (13)	73% (19)	73% (16)	75% (9)	43% (9)
**Reporting of handling of missing data, % (n)**	17% (28)	21% (15)	13% (12)	20% (4)	10% (3)	8% (1)	5% (1)	27% (7)	9% (2)	25% (3)	33% (7)
**Intention-to-treat analysis reported, % (n)**	42% (67)	53% (37)	33% (30)	70% (14)%	24% (7)	33% (4)	26% (5)	46% (12)	27% (6)	58% (7)	57% (12)
**Reporting of *P *values and 95% CI % (n)**	24% (38)	31% (22)	20% (18)	45% (9)	24% (7)	8% (1)	16% (3)	19% (5)	32% (7)	58% (7)	5% (1)

Only 17% (n = 28) of groups reported on how missing data were handled: 50% (n = 14) carried forward last values, 27% (n = 8) performed a complete case analysis, 13% (n = 4) used a fixed value imputation and 10% (n = 3) used more advanced methods, such as a regression model that also took into account patients with missing data, and a stratified imputation (one trial used two methods of handling missing data). Only 24% (n = 40) reported both *P *value and 95% CI. One or more primary outcomes were defined in 33% of trials (n = 53) but only 21% (n = 34) of the trials had a single primary outcome.

**Figure 1:**
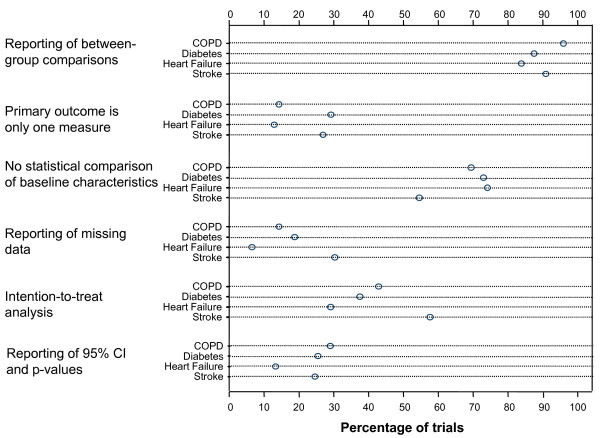
**Reporting of aspects of trial design, conduct and analysis**. The dot plot shows the proportion of trials in patients with chronic obstructive pulmonary disease (COPD) (49 trials), diabetes (48 trials), heart failure (31 trials) and stroke (33 trials) that reported on important aspects of trial design, conduct and analysis.

For three of the sources of bias we evaluated, the trials scored similarly across disease areas (Figure 1). For all four diseases, a single primary outcome was clearly defined in a similar proportion of trials, *P *value and 95% CI were reported in approximately 20% of trials, and between-group comparisons were conducted in approximately 90% of trials. Large differences between disease areas were present for the reporting of an ITT analysis (29 to 58% of trials), reporting on the handling of missing data (6 to 30% of trials) and not reporting on statistical comparisons of baseline characteristics (55 to 74% of trials). In the trials of drugs for heart failure, only 67% (n = 8) reported a between-group comparison, and this category of trials was also worse in terms of other sources of bias, whereas trials of drugs for COPD scored higher than drug trials of any other disease areas.

### Association of trial quality with type of intervention, year of publication and impact factor

Simple regression analysis (Table 2) showed that drug trials were significantly more likely than non-drug trials to clearly define one or more primary outcomes, to include an ITT analysis, and to avoid testing for differences in baseline characteristics. Defining one or more primary outcomes was significantly more likely after the year 2001. Finally, a high impact factor was associated with defining one or more primary outcomes, reporting of the handling of missing data, inclusion of *P *values and 95% CI, and use of an ITT analysis. In multivariate analyses (Table 3), drug trials were still more likely than non-drug trials to define one or more primary outcomes, to conduct an ITT analysis, and to avoid testing for differences in baseline characteristics. A more recent year of publication remained associated with defining one or more primary outcomes and the reporting of handling of missing data. A higher impact factor remained strongly associated with the reporting of one or more primary outcomes, a description of the handling of missing data, and the reporting of *P *values and 95% CI.

**Table 2:** Simple logistic regression to compare aspects of trial design, conduct and analysis by types of intervention, time before and after CONSORT (2001) and impact of journal

	Drug, % (n)	Non-drug, % (n)	**Odds ratio (95% CI)**(***P*-value**)	From 2001, % (n)	Before 2001,% (n)	Odds ratio (95% CI) (*P *value)	Odds ratio (95% CI) (*P *value) per increase of 1 unit in impact factor
Number of trials	70	91	-	72	67	-	161
Reporting of between- group comparisons	90% (63)	90% (82)	**0.98 **(0.35 to 2.80) (0.98)	96% (69)	91% (61)	**2.26 **(0.54 to 9.44) (0.26)	**1.03 **(0.97 to 1.11) (0.32)
Primary outcome reported	47% (33)	20% (18)	**4.06 **(2.02 to 8.14) (< 0.001)	53% (38)	21% (14)	**4.23 **(2.00 to 8.95) (< 0.001)	**1.05 **(1.02 to 1.09) (0.001)
Primary outcome is only one measure	31% (22)	13% (12)	**3.02 **(1.37 to 6.65) (0.006)	36% (26)	12% (8)	**4.17 **(1.73 to 10.06) (0.001)	**1.05 **(1.02 to 1.08) (0.002)
No statistical comparison of baseline characteristics	79% (55)	60% (55)	**2.40 **(1.18 to 4.88) (0.015)	68% (49)	70% (47)	**0.91 **(0.44 to 1.86) (0.79)	**1.03 **(1.00 to 1.08) (0.089)
Reporting of handling of missing data	21% (15)	14% (13)	**1.63 **(0.72 to 3.71) (0.24)	26% (19)	13% (9)	**2.31 **(0.96 to 5.55) (0.06)	**1.04 **(1.01 to 1.07) (0.021)
Intention-to-treat analysis reported	53% (37)	33% (30)	**2.28 **(1.20 to 4.33) (0.01)	53% (38)	39% (26)	**1.76 **(0.90 to 3.46) (0.10)	**1.03 **(1.00 to 1.06) (0.047)
Reporting of P values and 9% CI	30% (21)	19% (17)	**1.87 **(0.89 to 3.89) (0.10)	32% (23)	22% (15)	**1.63 **(0.76 to 3.47) (0.21)	**1.07 **(1.04 to 1.11) (< 0.001)

**Table 3:** Multiple logistic regression to assess the associations of types of intervention, time before and after CONSORT and impact of journal with aspects of trial design, conduct and analysis

	Drug versus non-drug	From 2001 versus before 2001	Per increase of 1 unit in impact factor
Reporting of between-group comparisons	**2.39 **(0.46 to 12.50) (0.30)	**2.73 **(0.63 to 11.90) (0.18)	**1.19 **(0.95 to 1.49) (0.14)
Primary outcome reported	**8.15 **(3.16 to 21.03) (< 0.001)	**8.46 **(3.18 to 22.49) (< 0.001)	**1.06 **(1.01 to 1.11) (0.010)
Primary outcome is only one measure	**4.19 **(1.68 to 10.46) (0.002)	**5.81 **(2.17 to 15.59) (< 0.001)	**1.04 **(1.01 to 1.08) (0.018)
No statistical comparison of baseline characteristics	**2.48 **(1.11 to 5.56) (0.027)	**0.97 **(0.46 to 2.03) (0.93)	**1.0 2 **(0.98 to 1.06) (0.30)
Reporting of handling of missing data	**1.79 **(0.74 to 4.32) (0.20)	**2.47 **(1.00 to 6.10) (0.049)	**1.03 **(1.00 to 1.06) (0.07)
Intention-to-treat analysis reported	**2.88 **(1.39 to 5.99) (0.005)	**2.02 **(0.99 to 4.15) (0.055)	**1.03 **(0.99 to 1.06) (0.11)
Reporting of P values and 95% CI (n)	**1.85 **(0.81 to 4.24) (0.15)	**1.83 **(0.79 to 4.22) (0.16)	**1.07 **(1.03 to 1.12) (< 0.001)

Data are odds ratios (95% CI) (*P *value)

### Reporting of subgroup analyses

A subgroup analysis of variables measured before randomization was reported in 27% (n = 43) trials; of these, only 23% (n = 10) reported an interaction test, whereas 77% reported either separate tests for each subgroup or tests for one subgroup only. Of the 43 trials reporting subgroup analyses, 81% (n = 35) reported a small number (< 5) of subgroups. In trials (n = 19) in which more than one significant subgroup effect was reported, 21% (n = 4) reported whether these effects were independent from other subgroup effects (that is, if interaction terms were still significant when other interaction terms were in the same regression model). Of the 34 trials that reported related outcomes, only 15% (n = 5) of trials found a consistent direction of the subgroup effects among closely related outcomes. Finally, of the forty-three trials that performed a subgroup analysis, 16% (n = 7) specified a hypothesis for a subgroup effect a priori but only two of these trials explained the rationale of their hypothesis by discussing prior evidence and biological plausibility. None of the trials pre-specified the direction of a potential subgroup effect.

## Discussion

### Main findings

We found in our survey of 161 chronic disease trials of widely prescribed therapies that an alarmingly large proportion of trials do not define a primary outcome, use naive methods to handle missing data (if at all), or do not use appropriate methods for subgroup analyses, or they test for baseline differences between groups. These findings indicate that a substantial proportion of trials are at risk for confounding or selection bias, or for not reporting the results in a way that would support clinical decision-making. Trials published after wide adoption of the CONSORT statement by journals in 2001 were significantly better in some aspects of trial quality, but factors other than the CONSORT statement may also have contributed to this improvement. Trials published in high-impact journals were significantly better at reporting both *P *values and 95% CI, and in specifying a primary outcome.

In all disease areas, most trials reported between-group comparisons, which is a promising sign that the problem of reporting within-group comparisons is not very prevalent in the literature on chronic disease. A greater problem is the still prevalent practice of reporting statistical comparisons of baseline characteristics, particularly in the stroke literature. It may be that in stroke-research investigators face a particular challenge of a heterogeneous patient population in which simple randomization or smaller trials may not yield balanced groups. However, statistical testing for differences in baseline characteristics does not provide appropriate guidance for adjustment for confounders. For the three aspects of definition of a primary outcome, handling of missing data and reporting of *P *values and 95% CI, the proportion of trials reporting these was low.

An interesting finding was that one-third of trials that specified a primary outcome actually defined more than one primary outcome. Many authors would argue against this practice because having several primary outcomes may lead to difficulties in interpretation or even selective outcome reporting. To prevent this would require a sample size that provides sufficient power for all primary outcomes. In addition, it may be difficult to choose means to control for confounding because the confounders could be different for different outcomes. However, the practice of multiple primary outcomes is currently supported by the European Medicines Agency (EMA), which requires industry trials on COPD to show efficacy for two co-primary outcomes [32]. Specifying co-primary outcomes may still be better than specifying none, but it will be interesting to see whether difficulties in interpretation of trials arise once trials following the EMA guidance are published.

## Results in the context of the literature

Several reviews have focused on the conventional aspects of trial quality, such as reporting of methods of masking, randomization or concealment of random allocation [2-4,6], but we are not aware of any other reviews in the area of chronic diseases that have analyzed all of the aspects of trial design and analysis that are particularly important for chronic disease trials [29]. A review of endocrinology trials that were published in 2005 and 2006 found that 34% of trials reported a clearly defined primary outcome, a result similar to what we found for the diabetes trials [33]. Another review focused on subgroup reporting, and found a similar extent of reporting of interaction tests as we did [34]. Bath *et al*. reviewed the stroke literature, and assessed conventional aspects of trial quality [35], and did not find publication in high-impact journals to be strongly associated with trial quality. Finally, a survey of more than 300 trials in COPD found, as we did, that adoption of the CONSORT statement improved the reporting of conventional aspects of trial design [1]. Overall, the results we found for each of the four clinical domains are similar to those of previous studies that were restricted to one disease area. This is important because it shows that our selection of trials, based on existing Cochrane reviews, seems to be a good representation of the trials in COPD, diabetes, heart failure and stroke, and thus allow for comparisons across these four disease areas.

### Strengths and limitations

The strengths of this study are the number and the diversity of the included trials, thus providing a broad overview of chronic disease trials of widely prescribed therapies. Hence, the quality of these trials is of great relevance to inform medical decision-making. Our survey goes beyond conventional aspects of internal validity. We believe that the aspects we assessed here, which are all referred to in the CONSORT statement, are closely related to the internal validity and interpretability of chronic disease trials [36,37]. We paid great attention to a rigorous data-extraction process and to standardized data collection in a secured database. A limitation of our study is that we did not assess whether the aspects of study design and reporting assessed in this paper affected the effect estimates. However, the relationship between reported aspects of trial quality and effect estimates is very challenging, and would require a different approach and study [6]. Finally, because only 17% of trials reported on the handling of missing data, we had only limited ability to investigate how the investigators handled missing data. Future studies should focus on the reporting of missing data and explore, based on more data, what methods (none, naive or advanced methods) investigators use to deal with this.

### Implications of our results for the design, reporting and interpretation of chronic disease trials

Our results show that current trials in patients with chronic disease are not optimally designed, conducted, analyzed and reported, and as a consequence, readers may often be left with uncertainty about the validity of the trials' findings. What can be done to improve the quality of trials in patients with chronic disease? Investigators carrying out clinical trials may not always know why certain features of trial design are important. We believe that a way forward would be not only to inform investigators what ought to be carried out and reported (as is laid out in the CONSORT statement) but also to have a better explanation of how the features of trial design help reduce bias. A clinical trial is no different from any epidemiological study; the primary concern should be minimization of confounding, selection bias and measurement error that lead to information bias.

There seems to be too little awareness of the problems of selection bias and missing data. A number of studies have found that many trials do not report or use inappropriate methods to conceal the random allocation [38]. Similarly, we found that many trials do not report on the handling of missing data and ways to deal with it. It is difficult to quantify the bias that results from different ways of (not) dealing with missing data because individual patient data from many different trials would be needed to investigate this. To improve reporting on missing data, editors and reviewers should require investigators to follow the CONSORT statement and report on their efforts to minimize bias from missing data and to report when they were unable to do so explicitly. The CONSORT statement includes all of the aspects of trial quality discussed here, and several studies, including ours, have shown that it was associated with the quality of reporting at least to some extent [1].

## Conclusion

From our survey of 161 randomized trials, we found that an alarmingly large proportion of chronic disease trials do not define a primary outcome, do not use appropriate methods for subgroup analyses, or use naive methods to handle missing data, if at all. As a consequence, biases are likely to be introduced in many trials on widely prescribed treatments in patients with chronic disease, and thus clinical decision-making based on these trials often may not be well informed. In addition to recommending wider adoption of the CONSORT statement, we suggest that investigators see clinical trials as any epidemiologic study in which particular attention needs to be paid to an optimum control for biases in accordance with established epidemiologic methods.

## Competing interests

All authors declare that they have no conflicts of interest or financial or other relationships that may influence or bias this work.

## Authors' contributions

MP conceived the study idea. DV, CB and MP developed the study protocol and database. DV, CB and MP extracted and checked the data. DV and MP analyzed the data. DV wrote the first draft of the manuscript. CB and MP read and critically revised the manuscript. All authors read and approved the final manuscript.

## Pre-publication history

The pre-publication history for this paper can be accessed here:

http://www.biomedcentral.com/1741-7015/9/73/prepub
